# Stage 2 Registered Report: Propositional Thought Is Sufficient for Imaginal Extinction as Shown by Contrasting Participants With Aphantasia, Simulated Aphantasia, and Controls

**DOI:** 10.1111/psyp.14756

**Published:** 2025-01-23

**Authors:** Merlin Monzel, Thomas Agren, Matthias Tengler, Jana Karneboge, Martin Reuter

**Affiliations:** ^1^ Department of Psychology University of Bonn Bonn Germany; ^2^ Department of Psychology Uppsala University Uppsala Sweden; ^3^ Department of Occupational Health, Psychology and Sports Science University of Gävle Gävle Sweden

**Keywords:** alexithymia, aphantasia, electrodermal activity, imaginal extinction, mental imagery, skin conductance response

## Abstract

Imaginal exposure is a standard procedure of cognitive behavioral therapy for the treatment of anxiety and panic disorders. It is often used when in vivo exposure is not possible, too stressful for patients, or would be too expensive. The Bio‐Informational Theory implies that imaginal exposure is effective because of the perceptual proximity of mental imagery to real events, whereas empirical findings suggest that propositional thought of fear stimuli (i.e., thinking about the stimuli without seeing them in the mind's eye) could be sufficient. To investigate whether mental imagery or propositional thought is crucial for the success of imaginal exposure, participants with the rare state of aphantasia (= absence of sensory mental imagery) and two control groups were subjected to a fear conditioning paradigm followed by imaginal exposure and a reinstatement procedure. During imaginal exposure, a control group (*N* = 30) stared at a bright screen to disrupt visual imagery by incoming luminance (=simulated aphantasia), while a second control group (*N* = 30) and participants with actual aphantasia (*N* = 30) kept their eyes closed. Results showed successful extinction in all groups, thus demonstrating that imaginal extinction is possible using propositional thought. Moreover, exploratory analyses revealed less subjective fear in people with aphantasia during the fear conditioning procedure, potentially due to similar mechanisms as in alexithymia, that is, a decoupling between physiological arousal and emotional experience.

## Impact statement

When contrasting people with aphantasia (= absence of mental imagery), simulated aphantasia, and controls in a fear conditioning paradigm with subsequent imaginal extinction, we found that visual imagery is not necessary for imaginal extinction to occur. Therefore, imaginal exposure therapy could also be effective for people with aphantasia.

## 1 Introduction

Exposure therapy is an effective psychological treatment for many anxiety disorders (Bandelow et al. 2015, 2018; Ougrin 2011). It is delivered either via in vivo exposure, where the patient is actually exposed to the fear stimuli, or via imaginative techniques, such as imaginal exposure, where the patient engages in mental imagery without facing the fear‐provoking stimuli, or a combination of both (Hackmann, Bennett‐Levy, and Holmes 2011). Mental imagery is defined as “representations and the accompanying experience of sensory information without direct external stimulus” (Pearson et al. 2015, 560) and is typically depicted as “seeing with the mind's eye, hearing with the mind's ear and so on” (Kosslyn, Ganis, and Thompson 2001, 635). A growing body of literature has provided evidence that mental imagery and sensory perception may stimulate similar brain regions which explains the “perception‐like” or “quasi‐sensory” character of the conscious mental image (Albers et al. 2013; Kosslyn, Ganis, and Thompson 2001; Marks 1973; Pearson 2019; Pearson et al. 2015). Therefore, it is not surprising that direct perception of phobic stimuli and mental imagery of phobic stimuli activate similar brain areas (Hoppe, Holmes, and Agren 2021).

Imaginal exposure has been an essential component of exposure therapy since its conception (Craske, Antony, and Barlow 2006; Wolpe 1958) and has been described to be as effective as in vivo exposure (Agren, Björkstrand, and Fredrikson 2017; Powers et al. 2010). In contrast to in vivo exposure, imaginal exposure offers certain advantages such as a controlled environment for confrontation (Mathews et al. 1976; Whelton 2004) and can be implemented even when in vivo confrontation would involve increased logistical or financial efforts as with, for example, aviophobia. In addition, the intrusive images that are characteristic of various anxiety and panic disorders are often not transferable to reality (e.g., catastrophizing thoughts), making in vivo exposure impossible. Imaginal extinction provides an experimental analog of imaginal exposure, in which a fear association is first established using differential fear conditioning and is extinguished afterward (e.g., Agren, Björkstrand, and Fredrikson 2017; Hoppe, Holmes, and Agren 2022). In this framework, extinction occurs through the repeated visualization of the fear‐inducing stimulus (CS+), omitting the presentation of the US (e.g., an electric shock).

Lang's Bio‐Informational Theory (Lang 1979) represents one of the first explanatory approaches concerning the underlying mechanisms of exposure treatments and states that anxiety‐related disorders are based on a specific pathological fear‐associated memory structure. According to the theory, mental imagery can stand in for real stimuli and events, because they are perceptually similar and trigger behavioral and physiological responses through the same fear‐associated memory structure. Emotion processing theory (EPT) (Foa and Kozak 1986; Foa and McLean 2016) incorporates Lang's idea as well as further refining assessable indicators for emotional processing as a fundamental element during therapy. At its core, it asserts that the outcome of exposure therapy is dependent on a sufficiently strong emotional activation as the main prerequisite, leading to the activation of the initial fear structure and secondly, the subsequent integration of incompatible information, resulting in an overall reduction of fear response (i.e., successful fear extinction). The vividness of visual imagery is considered to reflect the success of the emotional activation (Foa and Kozak 1986; Rauch et al. 2004). In this way, imaginal exposure became an integral part of prolonged exposure, a golden standard treatment for post‐traumatic stress disorder (PTSD) (Foa et al. 2019).

According to Lang's theory, the vividness of mental imagery is vital for imaginal exposure, and it would not expect arousal responses from verbal accounts of the stimuli without a visual imagery component. Indeed, results show that visual imagery reinforces positive and negative emotional states more strongly than verbal strategies (for review, see Holmes and Mathews 2010). In this vein, visual imagery is expected to be vital for the success of imaginal extinction and imaginal exposure. Yet, a meta‐analysis Rupp et al. (2017) came to the conclusion that there is no clear evidence that emotional activation is actually positively related to treatment outcome. Further studies provided similar evidence, that the within‐session strength of initial emotional activation does not necessarily predict therapeutic treatment outcome (Baker et al. 2010; Craske et al. 2014; Jaycox, Foa, and Morral 1998; Mota et al. 2015; Rauch et al. 2004). Similarly, it has not been clarified whether the effectiveness of imaginal exposure is based on mental imagery, particularly the emotional engagement studied by means of the vividness of the mental image, or whether propositional engagement leads to similar results. For instance, instead of visualizing the Eiffel Tower when asked about the most prominent landmark of Paris, people can simply know or belief that it is the Eiffel Tower without generating a mental image. Hence, imagination is not necessarily tied to mental imagery (Liao and Gendler 2011; Murphy 2022) and it should be examined whether mental imagery is a prerequisite for successful exposure therapy or if propositional thought is sufficient.

One alternative explanation holds that the observed symptom reduction during exposure therapy occurs due to non‐emotion‐based processes, such as inhibitory learning and reality testing (Rupp et al. 2017). In reality testing, extinction has the goal of correcting distorted or erroneous assumptions regarding the fear‐inducing situation or stimulus (Rupp et al. 2017). The focus is not on a subjectively perceived reduction of fear during exposure trials (as in the case of habituation‐based models of exposure therapy), but on changing the dysfunctional interpretation of the fear‐inducing situation (Craske et al. 2014). The aim is to expose oneself to the feared situation for as long as necessary to invalidate the truth of the dysfunctional expectations, which in turn promotes an adaptive or realistic approach to the feared situation, i.e. relying less on safety‐seeking behavior, and thus reduces the fear response. This mechanism is supported by the concept of inhibitory learning, according to which extinction aims to establish newly learned, secondary associations to replace the original pathological fear memory (Bouton 2002; Miller and Matzel 1988; Myers and Davis 2007). Thus, highly emotional arousal may not be decisive, but rather the omission of avoidance and safety strategies that previously prevented the examination of erroneous beliefs (Craske et al. 2014). Consequently, emotion‐triggering mental imagery (see Wicken, Keogh, and Pearson 2021) that is associated with higher levels of subjective distress in patients (Hoppe, Holmes, and Agren 2021; Mota et al. 2015; Rauch et al. 2004) would not be necessary for the effectiveness of imaginal exposure, which in turn would prevent therapy dropout (Coombs, Coleman, and Jones 2002; Eftekhari et al. 2020; Najavits 2015; Pascual‐Leone and Greenberg 2007).

Unfortunately, previous studies have not been able to conclusively test the necessity of mental imagery, as imaginal exposure is often carried out in conjunction with other psychological treatment elements (e.g., with verbal processing or in vivo exposure, for example, in Prolonged Exposure Therapy, Peterson, Foa, and Riggs 2019) and an isolated consideration of the mechanisms of imaginal exposure is thus only possible to a limited extent (e.g., Mota et al. 2015; Rauch et al. 2004). Hoppe, Holmes, and Agren (2022) first examined the influence of visual imagery on imagery extinction in isolation but came to ambiguous results. Although an overall interaction effect between stimulus (CS+/CS−) and trial (start, mid, and end) during imaginal extinction was found in the high vividness group but not in the low vividness group, this interaction was not due to an interaction between the vividness group and trial for the CS+. We argue that these ambiguous findings may be the result of limited variance in the vividness of visual imagery, as both the low‐vividness and the high‐vividness group had relatively average values in imagery vividness. The authors point out that “patients may only need to surpass a certain threshold of vividness for imaginal exposure to be effective” (Rauch et al. 2004), which is why greater group differences in imagery vividness should be targeted. Predestined for the dissociation of imagery and imagination are therefore people with aphantasia, describing a non‐clinical condition that is primarily manifested by the inability to generate voluntary sensory mental imagery, whereas the ability to think propositionally is preserved (Monzel et al. 2022; Zeman, Dewar, and Della Sala 2016, 2015). Wicken, Keogh, and Pearson (2021) showed that the physiological fear response when reading (i.e., imagining) scary stories was reduced in people with aphantasia, compared to a control group with no limitations in visual imagery. In a second condition, the authors demonstrated that this difference did not exist in direct visual viewing of anxiety‐provoking stimuli. The findings suggest that the reduced emotional physical response when imagining aversive scenarios is probably indicative of the lack of visual imagery rather than a generally reduced emotional or physical response.

Furthermore, to rule out the possibility that only higher order metacognitive processes are impaired in people with aphantasia and not actual visual imagery (i.e., visual imagery is still processed in early visual areas but does not become conscious), Pearson et al. (Pearson 2014; Pearson, Clifford, and Tong 2008; Pearson, Rademaker, and Tong 2011) developed a binocular rivalry task, with which the lack of visual imagery in people with aphantasia can be objectively determined by visual imagery priming (Keogh and Pearson 2018). In this task, participants are shown a binocular rivalry display consisting of red‐horizontal and blue‐vertical Gabor patterns while wearing blue‐red tinted anaglyph glasses. Normally, one of the two colored Gabor patterns becomes dominant by chance and suppresses the other one from awareness. However, imagining red or blue Gabor patterns beforehand leads to a higher probability of seeing the imagined Gabor pattern in the binocular rivalry display (=visual imagery priming) – at least when one was able to create visual imagery in the first place. Therefore, we will also use this task to validate group differences in visual imagery strength in our study. As a second safeguard against naturally occurring differences between people with aphantasia and controls apart from visual imagery ability and metacognitive awareness, we will implement two control groups. Sherwood and Pearson (2010) were able to show that incoming luminance signals are able to disrupt visual imagery. Therefore, control participants who undergo imaginal extinction with their eyes open while looking at a bright computer screen (luminance condition) should perform more similarly to individuals with aphantasia than control participants who undergo imaginal extinction with their eyes closed (no luminance condition). Less visual imagery with open eyes compared to closed eyes was also found by Caruso and Gino (2011). The control group in the luminance condition will be called the simulated aphantasia group in the following. Moreover, the same method could also be used in therapy if imaginal extinction is effective without visual imagery to block the patient's visual imagery and thus reduce distress and therapy dropout.

### 1.1 Aim of the Study

The present study aims to investigate the efficacy of imaginal extinction in (simulated) aphantasia to find out to what extent visual imagery^1^, rather than propositional thought, plays a role in fear reduction, and by extension, the treatment success in imagination‐based therapies. If the efficacy of imaginal extinction is found despite (simulated) aphantasia, this would have far‐reaching implications for the design of imaginal exposure. For instance, less vivid exposure (e.g., through imagery disruption procedures) could be used to reduce patients' distress and therapy dropout. If, however, in contrast to controls, no or less efficacy of imaginal extinction is found within people with (simulated) aphantasia, this would (a) be corroborating evidence for Lang's bio‐informational theory and its influence on EPT^2^ and (b) show that a standard procedure such as imaginal exposure might not apply to all patient groups. This would be particularly problematic as intrusive mental imagery is one of the most transdiagnostic aspects of mental disorders such as social anxiety disorder, generalized anxiety disorder, or PTSD (e.g., Hackmann, Bennett‐Levy, and Holmes 2011; Holmes and Mathews 2010) and, due to its intrusiveness, can also occur in people with aphantasia, as they are able to experience—at least partly—involuntary imagery (e.g., Zeman, Dewar, and Della Sala 2015). This would mean that the same intrusive images that develop in such a disorder cannot be voluntarily generated for exposure or imagery rescripting, making the disorders untreatable by these methods. PTSD, in particular, is an example of the significant impact of mental imagery in the form of involuntary recurrent images (i.e., flashbacks), which lead to great emotional distress through the reinforcement of pathological emotions (Iyadurai et al. 2020). Furthermore, Dawes et al. (2022) were able to show that a large sample of aphantasic individuals from a non‐clinical population scored similar to controls on the Post‐Traumatic Stress Disorder Checklist for DSM‐5 (PCL‐5, Weathers et al. 2013), although the symptom patterns differed between people with aphantasia and controls insofar as people with aphantasia showed lower frequency of memory intrusions and greater negative changes in cognition and mood. While there are no reliable empirical data on prevalence rates of PTSD among people with aphantasia, it might therefore also be possible for people with aphantasia to get PTSD. Thus, although prolonged exposure therapy has been shown to be highly effective in treating PTSD (Foa, Hembree, and Rothbaum 2007; Foa 2011; Powers et al. 2010), it might not be able to help clients with aphantasia. The assessment of the patient's vividness of visual imagery might then be a necessary part of therapy to be able to switch to alternative, non‐imagery‐based methods in case of aphantasia or less vivid imagery. Therefore, understanding the role of actual imagery in imaginal exposure would have wide‐reaching implications for the treatment of many disorders and the clinical psychology field in general. To assess the physiological fear response, we decided to use the skin conductance level in response to the CS+ and CS− as established in many studies before (e.g., Agren, Björkstrand, and Fredrikson 2017; Hoppe, Holmes, and Agren 2022).

H0: No interaction effect is found between the participant group (between‐subject; aphantasia vs. control vs. simulated aphantasia), stimulus (within‐subject; CS+ vs. CS−), and trial (within‐subject; start vs. mid vs. end) regarding the fear response in imaginal extinction measured by skin conductance response.

H1: An interaction effect is found between the participant group (between‐subject; aphantasia vs. control vs. simulated aphantasia), stimulus (within‐subject; CS+ vs. CS−), and trial (within‐subject; start vs. mid vs. end) regarding the fear response in imaginal extinction measured by skin conductance response. The fear response to CS+ decreases faster in controls than in people with aphantasia and simulated aphantasia, whereas the response to the CS− is the same for controls and people with aphantasia/simulated aphantasia.

Furthermore, reinstatement effects of imaginal extinction are assessed via a reinstatement procedure:

H0: No interaction effect is found between the participant group (between‐subject; aphantasia vs. control vs. simulated aphantasia), stimulus (within‐subject; CS+ vs. CS−), and trial (within‐subject; start vs. mid vs. end) regarding the fear response in reinstatement measured by skin conductance response.

H1: An interaction effect is found between the participant group (between‐subject; aphantasia vs. control vs. simulated aphantasia), stimulus (within‐subject; CS+ vs. CS−), and trial (within‐subject; start vs. mid vs. end) regarding the fear response in reinstatement measured by skin conductance response. The fear response to CS+ decreases faster in controls than in people with aphantasia and simulated aphantasia, whereas the response to the CS− is the same for controls and people with aphantasia/simulated aphantasia.

## 2 Method

### 2.1 Participants

Participants were recruited via the database of the Aphantasia Research Project Bonn (Monzel, Keidel, and Reuter 2021; Monzel, Vetterlein, and Reuter 2021) until 30 participants with aphantasia (VVIQ ≤ 23, cut‐off according to Zeman et al. 2020), 30 controls (VVIQ > 32, cut‐off according to Dance, Ipser, and Simner (2022); no luminance condition), and 30 controls with simulated aphantasia (VVIQ > 32; luminance condition) with successful fear conditioning (i.e., mean SCR in the CS+ trials—mean SCR in the CS− trials during fear acquisition > 0.10, root transformed and range corrected)^3^ were achieved. The sample size was determined via power analysis based on the effect size of the three‐way interaction between vividness group × stimulus × trial in Hoppe, Holmes, and Agren (2022) to obtain a power of 0.99 at an alpha level of 0.05. Participants are excluded when reporting current cardiologic, psychiatric, or neurological disorders, receiving psychological treatment or psychotropic medication within six months, age under 18 or over 60, pregnancy, drugs or excessive alcohol consumption, and non‐fluency in German.^4^ The participants were also asked not to consume alcohol for 24 h before their participation and not to take any analgesics (e.g., paracetamol, NSAIDs, or opioids) the week before. Violations of these requirements also led to exclusion from the study. Moreover, to rule out alterations in the transmission of the electric shocks, people with scars and tattoos on the right forearm, where the electric shocks were administered, were excluded from participation. Ethical approval of this study was given by the ethical board of the Institute of Psychology of the University of Bonn, Germany. All participants provided their written and informed consent. Participant compensation included participation in the draw of three vouchers of 50 € each as well as individual feedback about their visual imagery strength. Sample characteristics of the three experimental groups and statistics testing for group differences are given in Table 1. Samples were balanced regarding age, gender, and handedness.

**TABLE 1:** Descriptive statistics and tests for differences between groups in sociodemographic variables in the total sample.

	Aphantasia (*N* = 30)	Controls (*N* = 44)	Simulated aphantasia (*N* = 31)	Test statistic	*p*
Age					
*M*	32.67	26.45	25.19		
SD	10.82	8.61	6.86	6.39^a^	0.002
Gender					
Male (%)	40.0	25.0	25.8		
Female (%)	60.0	75.0	74.2		
Neither/Both (%)	0.0	0.0	0.0	2.22^b^	0.329
Handedness					
Left (%)	14.8	5.9	16.1	1.80^b^	0.772
Right (%)	81.5	94.1	80.6		
Both (%)	3.7	0.0	3.2		

^a^ *F*‐Test.

^b^ χ^2^‐Test.

### 2.2 Material

**FIGURE 1:**
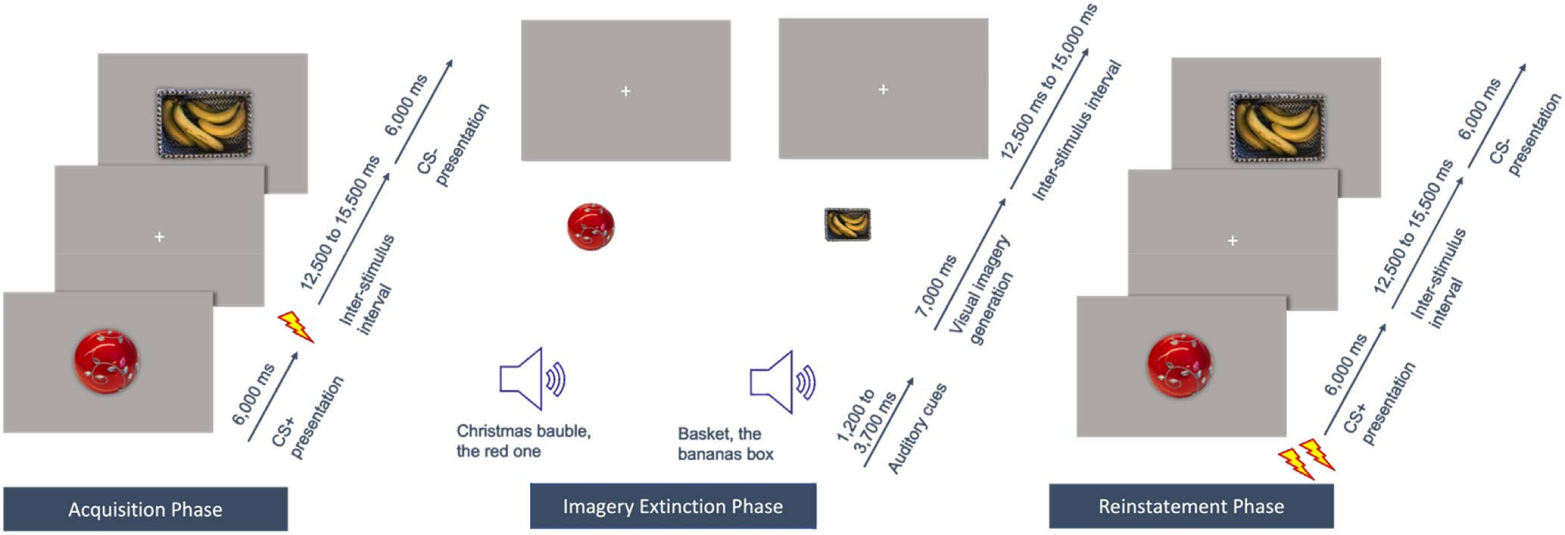
Exemplary sequential diagram of the three experimental phases with the Christmas bauble as CS+ and the banana box as CS−.

To maintain comparability, the same neutral stimuli were used as in Hoppe, Holmes, and Agren (2022). Since stimulus complexity did not influence the results in Hoppe, Holmes, and Agren (2022), only the complex stimuli were used to reduce the number of experimental trials, i.e. a Christmas bauble and a banana box (see Figure 1). The choice fell on the complex stimuli to create higher requirements for visual imagery, since not all people with aphantasia have a complete loss of visual imagery and might therefore be able to visualize at least very simple stimuli. Furthermore, complex stimuli are more natural and therefore more transferable to the therapeutic context.

### 2.3 Procedure

The procedure largely followed that of Hoppe, Holmes, and Agren (2022) and consisted of three experimental phases (see Figure 1). For economic reasons (since aphantasia is a relatively rare condition, many people with aphantasia had to travel long distances to participate in the study) and because the influence of visual imagery on fear extinction processes in general and not on long‐term fear memory was investigated, all three phases were carried out on 1 day. Nevertheless, to guarantee a certain degree of consolidation of fear and extinction memory, 1‐h breaks between the phases were ensured. The allocation of the stimuli to CS+ and CS− was counterbalanced. Experimenters were blinded to the group assignment of the participants.

At the beginning of each experimental phase, participants were asked to carefully pay attention to the screen and the audio instructions. They were also instructed that electric shocks may be administered during the experiment. The intensity of the electric shocks was determined by the participant prior to the start of the experimental phases via a step‐by‐step procedure until the shock was rated unpleasant, but tolerable. Participants were informed that the shock intensity would remain the same in all three phases. The actual experiment was carried out in a dark and quiet room. E‐prime 2.0 (Psychology Software Tools, Pittsburgh) was used to program the experiments. During all three experimental phases, skin conductance response (SCR) was assessed as the primary outcome measure for the physiological fear response. SCR was assessed via BIOPAC MP 160 (BIOPAC Systems, Goleta, CA) and two 8‐mm Ag/AgCl‐electrodes that were coated with isotonic electrolyte gel. The electrodes were attached to the hypothenar eminence of the participants' left hand. At the end of each experimental phase, participants were asked to rate the subjective fear they experienced during the respective phase (“How much fear did you experience during the last part of the experiment?”), the degree of expectancy that electric shocks would be administered (“How much did you expect to get electric shocks during the last part of the experiment?”), and their task compliance (“To what extent did you follow the instructions during the last part of the experiment?”) each on a scale ranging from 0 to 100. Each experimental phase consisted of a maximum of 20 trials (10 CS+, 10 CS−) that took about 8 min and 40 s. The stimulus sequence was randomized under the constraint that no more than two stimuli of the same type were presented after each other. Snacks and water were provided between the phases.

### 2.4 Phase 1—Fear Acquisition

The visual stimuli were displayed for 6 s on a 23′ computer screen with an inter‐stimulus interval that ranged from 12.5 to 15.5 s (*M* = 14.0 s) to avoid residual SCRs from previous trials. During the inter‐stimulus interval, a crosshair was presented. In total, each stimulus was shown ten times, with the CS+ being paired with a shock with a 100% reinforcement rate and the CS− never being paired with a shock. The shocks were administered using the BIOPAC MP 160 system's STMISOC module (BIOPAC Systems, Goleta, CA) through electrodes (EL500; BIOPAC Systems, Goleta, CA) prepared with isotonic electrolyte gel. They were delivered to the dorsal side of the right lower arm for 2 milliseconds, 250 milliseconds before the presentation of the CS+ ended.

### 2.5 Phase 2—Imaginal Extinction

Phase 2 started with visual imagery training to get the participants accustomed to the vividness scale (see Supporting Information of Hoppe, Holmes, and Agren 2022, for the training protocol). Moreover, to avoid participants not visualizing the visual stimuli due to memory effects (Bywaters, Andrade, and Turpin 2004) and to learn the link between the visual stimuli and the audio cues that describe the visual stimuli, the visual stimuli were presented for 15 s together with their matching audio cues immediately before the imaginal extinction procedure. During imaginal extinction, participants had to imagine the same visual stimuli as they had already encountered in the first experimental phase, prompted by the audio cues. Participants with aphantasia, as well as control participants in the no luminance condition, were instructed to keep their eyes closed at all times (while a black screen was displayed), whereas control participants with simulated aphantasia were instructed to keep their eyes open and to focus on the center of the screen, which was set to a bright white (luminance condition). To avoid extinction was linked to one specific audio stimulus rather than to the visual representation of the CS+, three different audio cues per visual stimulus were used in a pseudo‐randomized order, while the mental image stayed the same. The audio cues were presented for 1.2–3.7 s, followed by a 7.0 s period, in which the visual stimulus should be visualized. After that, a bell indicated an inter‐stimulus‐interval with randomized length that ranged from 12.5–15.5 s (*M* = 14.0 s) to allow time for the SCR to recover and ensure trial independence. The extinction always started with a CS−, which was excluded from the analysis, designed to catch novelty and orienting effects (Jiang and Greening 2021; Steinfurth et al. 2014). After this, each stimulus was imagined ten times and no shocks were delivered during the whole procedure. Directly after the imaginal extinction procedure, task specific‐vividness and subjective fear were assessed. Task‐specific vividness was assessed with a 5‐point Likert scale, adapted from the Vividness of Visual Imagery Questionnaire (VVIQ, Marks 1973, 1989), ranging from “no image at all, you only ‘know’ that you are thinking of an object” to “perfectly clear and as vivid as normal vision.”

### 2.6 Phase 3—Reinstatement

The reinstatement started with two unannounced and unpaired shocks to reactivate the fear memory (i.e., the association between CS+ and the electric shock). After that, no further shocks were applied. During reinstatement, participants were presented with the same visual stimuli they were exposed to during fearing acquisition and that they had visualized during imaginal extinction. Each stimulus was presented eight times. After the reinstatement procedure, participants completed the VVIQ (Marks 1973, 1989; German translation: Jungmann, Becker, and Witthöft 2022) and the trait version of the *Spielberger State–Trait Anxiety Inventory* (STAI‐Y) (Spielberger, Gorsuch, and Lushene 1970; German translation: Laux 1981).

### 2.7 Binocular Rivalry Task

The binocular rivalry task took place in the first break after the fear acquisition. After calibration of eye dominance (Pearson, Clifford, and Tong 2008), participants performed 32 binocular rivalry trials, in which they were asked to imagine either red‐horizontal or blue‐vertical Gabor patterns, before looking at a binocular rivalry display on a black background consisting of both. Since participants were wearing blue‐red tinted anaglyph glasses during these trials, only one type of Gabor pattern became dominant per trial. Participants indicated the type of Gabor pattern they saw by either clicking on blue‐vertical (1), perfectly mixed (2), or red‐horizontal (3). Mock rivalry displays, consisting of either blue or red Gabor patterns, were presented in 12.5% of the trials to detect participants' decisional biases.

### 2.8 Data Processing

The SCRs were filtered through a 0.05 Hz high‐pass hardware filter, and responses were calculated using trough to peak (max—min during a specific interval) using the neurokit2‐toolbox (Makowski et al. 2021). For fear acquisition and reinstatement, the intervals 1–5.75 s after visual stimulus onset were scored. The end of this interval coincided with the electric shock onset during fear acquisition, thus ensuring that SCRs reflect anticipatory fear, and were not triggered by the electric shocks. For imaginal extinction, the intervals 1–7 s after the end of the audio cue were scored. To test the learning criterion, all SCRs were root transformed and range corrected by dividing each response through the individual's maximum SCR across all experimental phases (Lykken 1972). For all further analyses, SCRs were root transformed and mean range corrected (Ben‐Shakar 1985). For each experimental phase, the single trials were partitioned into three bins, with the first three trials in “start”, the last three trials in “end”, and the remaining trials in “middle”. In accordance with Keogh and Pearson (2018), the priming score of the binocular rivalry task was computed by calculating the proportion of responses that matched the participants' visual imagery without including the mock trials.

### 2.9 Statistical Analyses

Participants with an average task compliance rating below 80% across all experimental phases were excluded from further analyses. Also, only participants that fulfilled the learning criterion for fear acquisition (i.e., mean SCR in the CS+ trials—mean SCR in the CS− trials during fear acquisition > 0.10, root transformed and range corrected) were planned to be included in the analyses. To examine if the likelihood of successful fear acquisition is already dependent on the vividness of visual imagery, a chi‐square test was conducted to assess the association of group assignment (people with aphantasia, controls) and fulfilling the learning criterion (yes, no). Differences in the strength of fear acquisition were assessed using an ANOVA with group assignment as an independent variable and the difference score of SCR in CS+ and CS− trials during fear acquisition as a dependent variable. Differences in the magnitude of shock intensity at subjective pain thresholds were analyzed by an additional ANOVA. A manipulation check for fulfilling/not fulfilling the learning criterion for fear acquisition was calculated by testing SCR in CS+ against SCR in CS− trials within designated learners and non‐learners (cf. Lonsdorf et al. 2019). To assess the robustness of the effects, all of the following analyses were repeated without any exclusion of participants.

Next, group assignment was validated via ANOVA by testing the VVIQ sum scores, the task‐specific vividness ratings in the fear extinction phase as well as the priming scores of the binocular rivalry task for differences between people with aphantasia, controls, and controls with simulated aphantasia. The association of dispositional and task‐specific vividness of visual imagery, as well as imagery strength in the binocular rivalry task, was examined using correlations. For these analyses, participants' binocular rivalry data was excluded when 50% of the mock trials were answered incorrectly. Furthermore, differences in trait anxiety measured by the STAI‐Y were assessed via ANOVA to account for any differences in trait anxiety in the interpretation of the main results. Task‐related subjective fear and electric shock expectancy were analyzed via ANOVA with experimental phase (fear acquisition, imaginal extinction, reinstatement) as within‐subject variable and group assignment (people with aphantasia, controls, simulated aphantasia) as between‐subject variable. Also, the association of trait anxiety, task‐related fear, and electric shock expectancy was assessed via correlations.

After that, hypotheses were tested via two separate ANOVAs with stimulus (CS+, CS−) and trial (start, mid, end) as within‐subject variables and group assignment (people with aphantasia, controls, simulated aphantasia) as between‐subject variables with SCR for imaginal extinction and reinstatement as the dependent variable. All statistical analyses were performed using JASP, version 0.16.2, to assess traditional statistics of null hypothesis significance testing as well as of Bayesian inferential methods using the default prior. When a result was significant, a Bayes Factor in favor of the alternative hypothesis was reported (*BF*_*10*_), otherwise, a Bayes factor in favor of the null hypothesis was reported (*BF*_*01*_). For more information on Bayesian inferential methods, see Schmalz et al. (2023). Whenever necessary, sphericity correction according to Greenhouse–Geisser was used.

## 3 Results

### 3.1 Recruitment

It was noted during recruitment that participants with aphantasia were less likely to acquire fear in the fear acquisition phase. Indeed, when tested later in the total sample, participants with aphantasia (60.0%) were 1.30 times less likely to be classified as learners than participants without aphantasia (78.1%), χ^2^(1) = 3.51, *p* = 0.061, *OR* = 2.38, although this difference was not significant. In the face of having to collect almost double the number of participants with aphantasia to reach our goal of 30 aphantasics with successful fear acquisition, we decided, in consultation with the editor, to stop data collection after we had collected at least 30 participants per group due to resource constraints. Because the recruitment of other groups continued until the recruitment of participants with aphantasia was completed, the final sample consisted of 30 participants with aphantasia, 44 controls, and 31 participants in the simulated aphantasia condition (*N* = 105). Participants in the simulated aphantasia group (*M* = 78.28, SD = 15.33) were less compliant than participants with aphantasia (*M* = 88.20, SD = 14.64), *t*(59) = 2.58, *p* = 0.012, *d* = 0.65, BF_10_ = 4.00, and controls (*M* = 90.58, SD = 11.09), *t*(51.33) = 3.82, *p* < 0.001, *d* = 0.94, BF_10_ = 173.68, possibly indicating that the incoming luminance actually affected their ability to imagine the stimuli and it was therefore too exhausting for them to follow the instructions to visualize, or simply indicating distraction due to their eyes being open in comparison to the other two groups. After applying our pre‐registered exclusion criteria for compliance and fear acquisition, the final sample would have consisted of 16 participants with aphantasia, 27 controls, and 15 participants in the simulated aphantasia condition, which would have considerably reduced our statistical power. Because of this, and the fact that fear acquisition was successfully produced also when calculated over all participants, we decided to drop these unusually strict criteria (Lonsdorf et al. 2017). However, an analysis using the subsample of participants who met these criteria can be found in the Supporting Information (see Table S2). Differences in the results between the samples are highlighted throughout the results section.

### 3.2 Manipulation Check: Fear Acquisition

For the manipulation check, two participants had to be excluded due to corrupted data (e.g., detached electrodes), leaving *N* = 103 (30 participants with aphantasia, 42 controls, and 31 participants in the simulated aphantasia condition), still achieving the a priori calculated power. Fear acquisition was successful as there was a strong main effect of stimulus over all three groups, *F*(1, 100) = 148.58, *p* < 0.001, η_p_^2^ = 0.60, BF_10_ = 1.40 × 10^19^. There was no group × stimulus × trial interaction effect, *F*(4, 200) = 0.37, *p* = 0.831, η_p_^2^ < 0.01, BF_01_ = 31.60, or trial × group effect, *F*(3.02, 150.99) = 0.10, *p* = 0.962, η_p_^2^ < 0.01, BF_01_ = 44.75. However, over all trials, there was a near‐significant stimulus × group effect, *F*(2, 100) = 2.44, *p* = 0.092, η_p_^2^ = 0.05, BF_01_ = 1.31, but when specifically analyzing the end of acquisition, no differences remained, *F*(2,100) = 0.86, *p* = 0.425, η_p_^2^ = 0.02, BF_01_ = 5.61, indicating a similar fear acquisition in all groups.

A look at the subjective pain threshold showed no significant differences between the shock intensity either, *F*(2, 100) = 1.58, *p* = 0.211, BF_01_ = 3.16, indicating that, on average, the same shock intensity was used for participants with aphantasia (*M* = 38.70 mA, SD = 12.97 mA), controls (*M* = 33.87 mA, SD = 16.44 mA), and participants in the simulated aphantasia condition (*M* = 31.94 mA, SD = 15.94 mA). The fear acquisition was stronger within designated learners than within participants not fulfilling the fear acquisition learning criterion as indicated by an interaction effect between stimulus (CS+ vs. CS−) and group (designated learners vs. participants not fulfilling the learning criterion), *F*(1, 101) = 64.11, *p* < 0.001, η_p_^2^ = 0.39, BF_10_ = 3.35 × 10^9^. However, the participants not fulfilling the criterion for fear acquisition also separated CS+ and CS−, *t*(27) = 3.08, *p* = 0.005, *d* = 0.58, BF_10_ = 8.71, demonstrating fear acquisition.

### 3.3 Manipulation Check: Group Assignment

**FIGURE 2:**
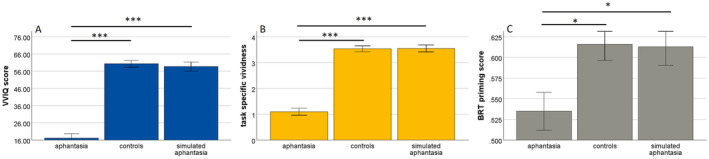
Differences in visual imagery ability between the groups measured by (A) the vividness of the visual imagery questionnaire, (B) task‐specific vividness, and (C) the priming score in the binocular rivalry task. ***p < 0.001, *p < 0.05.

Groups differed significantly in the VVIQ sum scores, *F*(2, 102) = 222.37, *p* < 0.001, η_p_^2^ = 0.81, BF_10_ = 2.34 × 10^34^, the task‐specific vividness, *F*(2, 101) = 112.50, *p* < 0.001, η_p_^2^ = 0.69, BF_10_ = 1.24 × 10^23^, as well as the priming score of the binocular rivalry task, *F*(2, 100) = 4.29, *p* = 0.016, η_p_^2^ = 0.08, BF_10_ = 2.71. Post hoc tests revealed that these effects were based on the differences between the participants with aphantasia and the other two groups (see Figure 2). Correlational analyses revealed significant associations between the priming score in the binocular rivalry task and the VVIQ, *r*(103) = 0.27, *p* = 0.005, BF_10_ = 5.73, the task‐specific vividness and the VVIQ, *r*(104) = 0.88, *p* < 0.001, BF_10_ = 7.61 × 10^31^, as well as the priming score in the binocular rivalry task and task‐specific vividness, *r*(102) = 0.20, *p* = 0.040, BF_10_ = 0.99 (see Figure S1).^5^ Thus, the three mental imagery indices showed convergent validity and participants with aphantasia had less vivid imagery and less imagery strength than participants without aphantasia. No other results were found in the more homogenous subsample analyzed in the supplemental material.

### 3.4 Manipulation Check: Detectable Skin Conductance Responses to Mental Imagery

For the purpose of demonstrating that mental imagery successfully evoked skin conductance responses during imaginal extinction, we adopted a somewhat conservative stance on a detectable skin conductance response and counted how many responses exceeded 0.05 μS during the imaginal extinction. There were 705 out of 950 (74.2%) responses to CS+ and 585 out of 950 (61.6%) responses to CS−, the difference probably reflecting the attenuating responses to CS−. Thus, the mental imagery stimuli during extinction did evoke SCRs. Moreover, there was no significant difference between the groups in number of responses to either CS+, *F*(2, 92) = 2.18, *p* = 0.119, BF_01_ = 1.76, or CS−, *F*(2,92) = 2.24, *p* = 0.112, BF_01_ = 1.68. Aphantasics had a mean of 6.68 CS+ responses and 5.90 CS− responses exceeding 0.05 μS, demonstrating that even among aphantasics the mental processes resulting from the stimulus instruction evoked SCRs.

### 3.5 Fear Extinction

**FIGURE 3:**
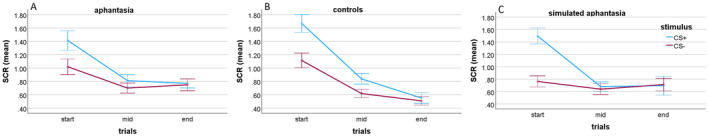
Square root transformed, mean range corrected SCR for (A) participants with aphantasia, (B) controls, and (C) participants in the simulated aphantasia condition across the fear extinction phase (±1 SEM).

For the fear extinction analyses, ten participants had to be excluded due to corrupted data, leaving *N* = 95 (28 participants with aphantasia, 41 controls, and 26 participants in the simulated aphantasia condition). Results showed a significant stimulus × trial interaction effect, *F*(1.62, 148.94) = 19.61, *p* < 0.001, η_p_^2^ = 0.18, BF_10_ = 3.63 × 10^6^, indicating an overall successful fear extinction procedure. T‐tests confirmed that participants differed in response between CS+ and CS− in the beginning, *t*(94) = 7.15, *p* < 0.001, *d* = 0.73, BF_10_ = 5.15 × 10^7^, and middle, *t*(94) = 4.61, *p* < 0.001, *d* = 0.47, BF_10_ = 1315.96, but not at the end, *t*(94) = 1.00, *p* = 0.321, BF_01_ = 5.44. Crucially, there was neither a main effect of group, *F*(2, 92) = 0.68, *p* = 0.509, η_p_^2^ = 0.02, BF_01_ = 7.70, nor a stimulus × group, *F*(2, 92) = 0.85, *p* = 0.429, η_p_^2^ = 0.02, BF_01_ = 8.73, or group × stimulus × trial interaction effect, *F*(3.24, 148.94) = 1.30, *p* = 0.278, BF_01_ = 5.51, indicating no differential fear extinction between the groups (see Figure 3). As a follow‐up, groups were also analyzed individually. The aphantasia group showed a significant stimulus × trial effect, *F*(2, 54) = 3.70, *p* = 0.031, η_p_^2^ = 0.12, BF_10_ = 1.99, separating CS+ and CS− at the beginning, *t*(27) = 3.13, *p* = 0.004, *d* = 0.59, BF_10_ = 9.69, and middle, *t*(27) = 2.42, *p* = 0.022, *d* = 0.46, BF_10_ = 2.35, but not at the end, *t*(27) = 0.76, *p* = 0.451, *d* = 0.14, BF_01_ = 3.82. Controls also showed a stimulus × trial, *F*(1.55, 61.88) = 6.91, *p* = 0.004, η_p_^2^ = 0.15, BF_10_ = 50.04, separating CS+ and CS− at beginning, *t*(40) = 4.49, *p* < 0.001, *d* = 0.70, BF_10_ = 388.54, and middle, *t*(40) = 3.58, *p* < 0.001, *d* = 0.36, BF_10_ = 32.34, but not at the end, *t*(40) = 1.08, *p* = 0.289, *d* = 0.17, BF_01_ = 3.46. Lastly, the simulated aphantasia group showed a stimulus × trial, *F*(1.59, 39.65) = 10.58, *p* < 0.001, η_p_^2^ = 0.30, BF_10_ = 1026.33, separating CS+ and CS− at beginning, *t*(25) = 4.86, *p* < 0.001, *d* = 0.95, BF_10_ = 472.21, but not at middle, *t*(25) = 1.70, *p* = 0.102, *d* = 0.33, BF_01_ = 1.37, or at the end, *t*(25) = 0.01, *p* = 0.991, *d* < 0.01, BF_01_ = 4.83. Thus, all groups exhibited fear extinction behavior. Returning to the main ANOVA, there was a trial × group interaction effect, *F*(2.85, 131.22) = 3.90, *p* = 0.012, η_p_^2^ = 0.08, BF_10_ = 8.18, which was based on a stronger general habituation for controls as compared to the participants in the simulated aphantasia condition, *F*(1.49, 96.70) = 7.96, *p* = 0.002, η_p_^2^ = 0.11, BF_10_ = 54.31, possible dependent on the less compliant participants in the simulated aphantasia condition. This hypothesis is in line with the interaction effect disappearing in the more homogenous subsample analyzed in the supplemental material. Moreover, a significant main effect of stimulus, *F*(1, 92) = 53.36, *p* < 0.001, η_p_^2^ = 0.37, BF_10_ = 1.45 × 10^7^, and a significant main effect of trial were found, *F*(1.43, 131.22) = 85.92, *p* < 0.001, η_p_^2^ = 0.48, BF_10_ = 6.59 × 10^25^, indicating that the fear response was smaller for the CS− and declined for both, the CS+ and CS−. The descriptive statistics for all experimental phases can be found in the Supporting Information (see Table S1).

### 3.6 Reinstatement

**FIGURE 4:**
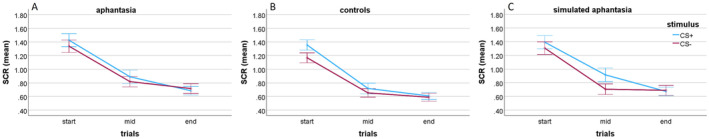
Square root transformed, mean range corrected SCR for (A) participants with aphantasia, (B) controls, and (C) participants in the simulated aphantasia condition across the reinstatement phase (±1 SEM).

For the reinstatement analyses, 14 participants had to be excluded due to corrupted data, leaving *N* = 91 (26 people with aphantasia, 40 controls, and 25 participants in the simulated aphantasia condition). Results showed a near‐significant stimulus × trial interaction effect, *F*(2, 176) = 2.40, *p* = 0.094, η_p_^2^ = 0.03, BF_01_ = 2.14. However, additional t‐tests showed that participants differed in response between CS+ and CS− in the beginning, *t*(90) = 2.73, *p* = 0.008, *d* = 0.29, BF_10_ = 3.72, and in the middle, *t*(90) = 2.04, *p* = 0.045, *d* = 0.21, BF_01_ = 1.20, but not at the end of the reinstatement, *t*(90) = 0.14, *p* = 0.885, *d = 0.02*, BF_01_ = 8.54. Thus, a return of fear effect was noted at the start of the reinstatement, which later faded. There was neither a significant main effect of group, *F*(2, 88) = 1.83, *p* = 0.168, η_p_^2^ = 0.04, BF_01_ = 2.35, nor a trial × group, *F*(3.38, 148.63) = 0.14, η_p_^2^ < 0.01, *p* = 0.949, BF_01_ = 46.44, stimulus × group, *F*(2, 88) = 0.39, *p* = 0.680, η_p_^2^ < 0.01, BF_01_ = 14.54, or group × stimulus × trial interaction effect, *F*(3.98, 174.95) = 0.63, η_p_^2^ = 0.01, *p* = 0.638, BF_01_ = 14.41, indicating no differential reinstatement between the groups (see Figure 4). In addition, a significant main effect of stimulus, *F*(1, 88) = 8.32, *p* = 0.005, η_p_^2^ = 0.09, BF_10_ = 2.71, and a significant main effect of trial were found, *F*(1.69, 148.63) = 154.06, *p* < 0.001, η_p_^2^ = 0.64, BF_10_ = 1.20 × 10^38^, indicating a return of fear effect and that responses for both stimuli declined during the reinstatement procedure. No other results were found in the more homogenous subsample analyzed in the supplemental material.

### 3.7 Trait Anxiety, Situational Fear, and Shock Expectancy

**FIGURE 5:**
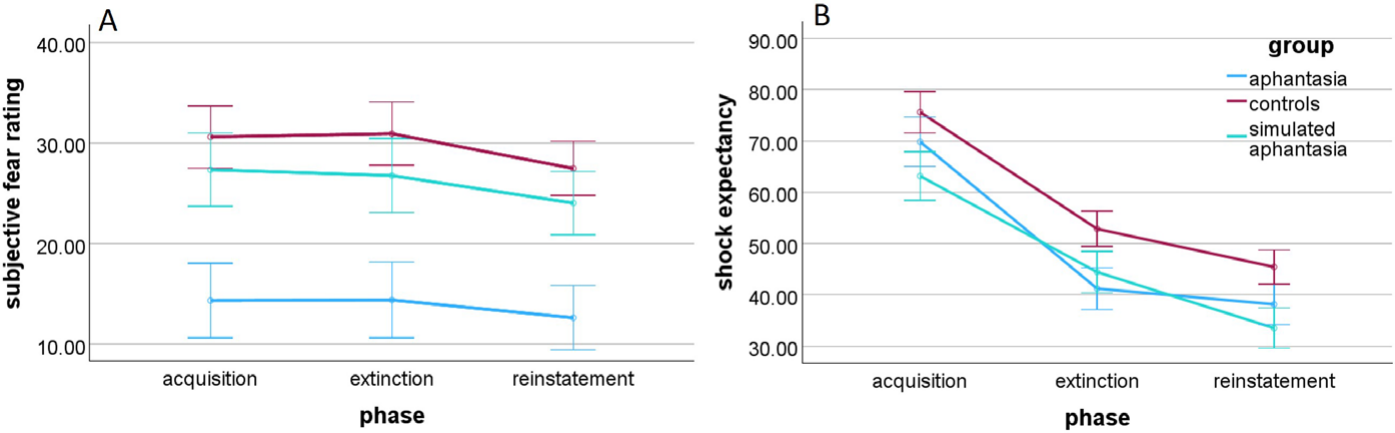
(A) Subjective fear ratings and (B) shock expectancies across fear acquisition, fear extinction, and reinstatement per group (±1 SEM).

Participants with aphantasia (*M* = 2.21, SD = 0.48), controls (*M* = 2.25, SD = 0.37), and participants in the simulated aphantasia condition (*M* = 2.12, SD = 0.46) did not differ significantly in trait anxiety measured by the STAI‐Y, *F*(2, 102) = 0.84, *p* = 0.433 BF_01_ = 5.63. Regarding the situational fear during the experimental procedure, there was a significant main effect of group, *F*(2, 101) = 7.62, *p* < 0.001, η_p_^2^ = 0.13, BF_10_ = 37.51, indicating that participants with aphantasia (*M* = 13.78, SD = 14.45) experienced less fear than controls (*M* = 30.00, SD = 19.47), *t*(72) = 3.83, *p*_*holm*_ < 0.001, *d* = 82, BF_10_ = 110.63, and participants in the simulated aphantasia condition (*M* = 26.03, SD = 16.94), *t*(60) = 2.74, *p*_*holm*_ = 0.014, *d* = 0.63, BF_10_ = 10.73. However, there was no significant difference between controls and participants in the simulated aphantasia condition, *t*(73) = 0.88, *p*_*holm*_ = 0.380, *d* = 0.19, BF_01_ = 2.88 (see Figure 5A).^6^ In the more homogeneous subsample analyzed in the Supporting Information, the difference between participants with aphantasia and participants in the simulated aphantasia condition was also not significant, most likely due to lower statistical power (*p* = 0.086). Finally, there was neither a main effect of the experimental phase, *F*(2, 202) = 2.15, *p* = 0.119, BF_01_ = 3.31, nor an interaction effect between the group and experimental phase, *F*(4, 202) = 0.08, *p* = 0.987, BF_01_ = 44.90.

An additional repeated‐measures ANOVA revealed a significant main effect of the experimental phase for shock expectancy, *F*(2, 202) = 76.31, *p* < 0.001, η_p_^2^ = 0.43, BF_10_ = 1.14 × 10^23^, showing a linear trend, *F*(1, 101) = 125.57, *p* < 0.001, η_p_^2^ = 0.55, indicating that the shock expectancy declined across the experimental phases (see Figure 5B). Moreover, there was a main effect of group, *F*(2, 101) = 3.69, *p* = 0.029, η_p_^2^ = 0.07, BF_10_ = 1.23, with controls (*M* = 58.69, SD = 17.22) showing higher shock expectancy than participants in the simulated aphantasia condition (*M* = 49.73, SD = 21.17), *t*(73) = 2.57, *p*_*holm*_ = 0.035, *d* = 0.47, BF_10_ = 15.15, probably due to a higher cognitive load in the simulated aphantasia condition that diverted the attention from the possible shocks. However, there was no difference between participants with aphantasia (*M* = 47.03, SD = 17.00) and controls, *t*(72) = 1.69, *p*_*holm*_ = 0.188, BF_01_ = 0.92, BF_01_ = 0.69, nor between the participants with aphantasia and participants in the simulated aphantasia condition, *t*(60) = 0.69, *p*_*holm*_ = 0.490, BF_01_ = 5.11. Moreover, there was no interaction effect between the experimental phase and group, *F*(4, 202) = 0.62, *p* = 0.647, BF_01_ = 21.58, indicating no different shock expectancy in each experimental phase dependent on the group. No other results were found in the more homogenous subsample analyzed in the Supporting Information.

### 3.8 Exploratory Analyses

In the light of a recent review article stating that mental imagery may be part of an inwardly focused cognitive style, which includes mental imagery, emotion processing, and interoceptive attention (Kvamme et al. 2024) as well as another recent paper showing that people with aphantasia were more often classified as alexithymic than controls (Monzel et al. 2024), we decided to match data from the Beck Anxiety Inventory (BAI; Beck et al. 1988) which was available for a part of the present sample. Our aim was to find evidence of a decoupling between subjective fear experience and physiological arousal in participants with aphantasia, as it is proposed for people with alexithymia (Luminet et al. 2004; Stone and Nielson 2001). While the BAI is not explicitly a measure of physiological anxiety symptoms, in contrast to the STAI, 16 out of 21 items mention physiological symptoms such as heart pounding, hands trembling, feeling hot, numbness or tingling, and many more. According to the BAI, people with aphantasia (*N* = 26, *M* = 29.04, SD = 9.57) reported less symptoms of anxiety than controls (*N* = 50, *M* = 34.28, SD = 11.02), *t*(74) = 2.06, *p* = 0.043, *d* = 0.49, BF_10_ = 1.47, although no objective differences in physiological arousal were found across the experimental phases.

## 4 Discussion

Our study aimed to investigate the efficacy of imaginal extinction in aphantasia and simulated aphantasia to find out to what extent imaginal exposure (Craske, Antony, and Barlow 2006; Wolpe 1958) is dependent on visual mental imagery rather than propositional thought. For this, we applied a standard fear conditioning paradigm to people with aphantasia, simulated aphantasia, and controls (cf. Hoppe, Holmes, and Agren 2022). A manipulation check showed that all groups acquired fear, using similar shock intensities. A second manipulation check validated the group assignment, showing that participants with aphantasia had less vivid visual imagery than people without aphantasia, both dispositionally and task‐specifically. All groups displayed fear extinction behavior, showing significant stimulus × trial effects, separating CS+ and CS− at the beginning of extinction, but not at the end. Thus, the present data demonstrate that visual imagery is not necessary for imaginal extinction to occur. Moreover, all groups displayed a return of fear effect in the reinstatement phase, and again, no group effects were found. Thus, participants with aphantasia not only showed short‐term effects of imaginal extinction, but they did also not differ from the control group or the simulated aphantasia group in the long‐term effects (or rather longer‐term effects, since all phases were conducted one the same day) of imaginal extinction, indicating that propositional thought is sufficient for imaginal extinction to occur. As imaginal extinction is partly constructed as an experimental model of imaginal exposure (Agren, Björkstrand, and Fredrikson 2017), this suggests that vivid mental imagery may also not be necessary for imaginal exposure. However, additional tests should be conducted in clinical practice to firmly conclude whether our results can be generalized to imaginal exposure, as the validity of single fear conditioning studies for real clinical practice is debatable (for further information, see Scheveneels et al. 2021).

### 4.1 Trait Anxiety, Situational Fear, and Shock Expectancy

Regarding shock expectancy, the expectancy of the US decreased in all experimental groups from acquisition to extinction, and from extinction to reinstatement, showing that the participants became aware that the association between the CS+ and the US was removed after fear acquisition. Participants with aphantasia experienced less fear across all phases of the fear conditioning paradigm compared to the other two groups. This effect was mirrored by results on BAI (Beck et al. 1988), where participants with aphantasia scored lower than controls, but not with STAI‐Y (Spielberger, Gorsuch, and Lushene 1970). In contrast to the STAI‐Y (Spielberger, Gorsuch, and Lushene 1970), the BAI assesses more physiological anxiety symptoms (e.g., feeling hot or shaky). Hence, a possible hypothesis is that aphantasics' subjective fear is less impacted by physiological responses, which is in line with the lower self‐reported fear during the fear conditioning paradigm, in spite of the fact that the physiological responses did not differ between aphantasic individuals and controls. Of note, a similar mechanism is proposed for alexithymia, in which a decoupling of emotional experience and physiological arousal is assumed (Luminet et al. 2004; Stone and Nielson 2001). Moreover, people with alexithymia report less vivid visual imagery than people without alexithymia (Campos, Chiva, and Moreau 2000; Mantani et al. 2005) and vice versa (Jungmann, Becker, and Witthöft 2022; Monzel et al. 2024). Thus, a similar mechanism can be proposed for aphantasia and alexithymia, for example, perhaps a strongly externally oriented thinking style (Monzel et al. 2024) leads to the inhibition of internally‐orientated thinking such as monitoring one's own emotional state or interoceptive evaluation (Kvamme et al. 2024). This could manifest in less vivid visual imagery and lower awareness of physiological arousal in people with aphantasia (Monzel, Keidel, and Reuter 2023; Wicken, Keogh, and Pearson 2021) as well as alexithymia (Nemiah, Freyberger, and Sifneos 1976; Taylor and Bagby 2021) and thus, in an underestimation one's own emotional state. However, possible interactions between physiological arousal and fear that are common to both alexithymia and aphantasia is a topic for future studies.

### 4.2 Strengths and Weaknesses of the Present Study Design

Overall, the present study was able to show that visual mental imagery is not a prerequisite for imaginal extinction to occur. Moreover, our study corroborates Holmes and Mathews' (2010) emotional amplification theory, which proposes that mental imagery amplifies emotional responses, but only regarding subjective fear (Monzel, Keidel, and Reuter 2023). Therefore, aphantasic individuals might not only profit from imaginal exposure but they might also be less distressed by its content (Hoppe, Holmes, and Agren 2021; Mota et al. 2015; Rauch et al. 2004) which could ultimately lead to less therapy drop‐out and therefore more favorable results (Coombs, Coleman, and Jones 2002; Eftekhari et al. 2020; Najavits 2015; Pascual‐Leone and Greenberg 2007).

Our design has some important strengths: First, we were able to validate the group assignment of people with and without aphantasia not only via self‐report but also via an objective visual imagery task, that is, the binocular rivalry task by Keogh and Pearson (2018). With this, the limitation of the limited variance in the vividness of visual imagery in the sample of Hoppe, Holmes, and Agren (2022) could be resolved. Second, different measures of dispositional and task‐specific fear were able to differentiate between physiological fear response and subjective emotional response, leading to a new hypothesis regarding the mechanisms of a weaker emotional experience in people with aphantasia. As was to be expected, the simulated aphantasia group did not report less subjective fear, as only the ability to generate mental images was manipulated and not any dispositional differences between controls and participants in the simulated aphantasia condition. Therefore, this third group proved to be valuable. Furthermore, differences in the compliance score suggested a successful manipulation of mental imagery using incoming luminance (Sherwood and Pearson 2010), but could also be the result of more distraction caused by their open eyes. Finally, although we dropped our unusually strict learning criteria for fear acquisition (Lonsdorf et al. 2017) and compliance to achieve the statistical power calculated in our a priori power analysis, a reanalysis with the smaller sample including only participants who met these criteria showed the same results (see Supporting Information). Thus, we believe the possible impact of potential sampling biases due to differences in fear acquisition and compliance is small. A limitation of this study is that, due to time constrains, the memories were not fully consolidated between experimental phases. Thus, our extinction and reinstatement effects may not be altogether generalizable to fully consolidated fear and extinction memories.

## 5 Conclusion

Within our study, visual mental imagery was not necessary for imaginal extinction to occur. Moreover, people with aphantasia experienced less subjective fear across all experimental phases, although showing the same physiological response. Thus, our data suggests that imaginal exposure could also be applicable to people with aphantasia, and even be less stressful for them. However, further studies are necessary to firmly conclude whether our results can be generalized to clinical practice.

## Author Contributions

**Merlin Monzel:** conceptualization, data curation, formal analysis, methodology, project administration, visualization, writing – original draft, writing – review and editing. **Thomas Agren:** data curation, formal analysis, methodology, validation, writing – review and editing. **Matthias Tengler:** investigation. **Jana Karneboge:** investigation, writing – review and editing. **Martin Reuter:** resources, supervision, writing – review and editing.

## Conflicts of Interest

The authors declare no conflicts of interest.

## Supporting information


Data S1.

## Data Availability

The data that support the findings of this study are openly available in the OSF at https://osf.io/fku9n/?view_only=6864c9c094cf492c835dfd58ce99cd3a.

## Endnotes

^1^
Although most people with aphantasia are affected in more than visual imagery (Dawes et al. 2022) and different sensory modalities of mental imagery are highly correlated (e.g., Jungmann, Becker, and Witthöft 2022), our study design only checks for visual imagery. However, since other sensory retrieval processes are theorized to be organized similarly (Blomkvist 2022), a generalizability to other sensory modalities seems likely.

^2^
It is important to mention that EPT itself does not inherently imply that emotional activation has to result from visual imagery. Therefore, in the framework of the EPT imaginal extinction might also work when emotional activation is achieved otherwise.

^3^
This cut‐off was obtained by calculating the critical difference which indicates whether two values differ from each other significantly or can be explained by random influences (e.g., due to test unreliability). Reliability (*α* = 0.88) and standard deviation of the SCR difference score (SD = 0.13) were obtained from Hoppe, Holmes, and Agren (2022). Alpha level was 0.05, right‐tailed.

^4^
All instructions and questionnaires were presented in German. To ensure international comprehensibility, the corresponding information in this article is presented in English.

^5^
Differences in degrees of freedom are due to two missing values in the binocular rivalry task and one missing value in the VVIQ.

^6^
The main effect of group remained significant when adding age as a covariate, *F*(2, 100) = 4.99, *p* = 0.009, η_p_^2^ = 0.09, BF_10_ = 7.77.
