# Supplementary material for: Stage 2 Registered Report: Propositional Thought Is Sufficient for Imaginal Extinction as Shown by Contrasting Participants With Aphantasia, Simulated Aphantasia, and Controls
Source: Psychophysiology. 2025 Jan 23;62(1):e14756. doi: 10.1111/psyp.14756 (PMC11755224; doi:10.1111/psyp.14756)
Supplement: Supplementary file 1 — Data S1. [file PSYP-62-e14756-s001.docx]

# Supplemental material

## Additional tables

*Table S1.* Root-transformed mean range corrected SCR to the CS+ and CS- across all experimental procedures (total sample).

|  | Trial bin^1^ | | | | | | | |
| --- | --- | --- | --- | --- | --- | --- | --- | --- |
|  | Start | |  | Mid | |  | End | |
|  | CS+ | CS- |  | CS+ | CS- |  | CS+ | CS- |
| **Total** |  |  |  |  |  |  |  |  |
| Acquisition (*n* = 103) | 1.62 (.06) | 1.27 (.05) |  | 1.44 (.05) | .86 (.03) |  | 1.39 (.05) | .81 (.04) |
| Extinction (*n* = 95) | 1.49 (.06) | 1.08 (.05) |  | .85 (.04) | .70 (.03) |  | .70 (.04) | .67 (.04) |
| Reinstatement (*n* = 91) | 1.39 (.05) | 1.25 (.05) |  | .82 (.05) | .72 (.04) |  | .65 (.32) | .65 (.04) |
|  |  |  |  |  |  |  |  |  |
| **Aphantasia** |  |  |  |  |  |  |  |  |
| Acquisition (*n* = 30) | 1.48 (.09) | 1.24 (.08) |  | 1.29 (.08) | .86 (.06) |  | 1.28 (.07) | .80 (.05) |
| Extinction (*n* = 28) | 1.45 (.11) | 1.19 (.09) |  | .88 (.06) | .74 (.05) |  | .78 (.06) | .74(.07) |
| Reinstatement (*n* = 26) | 1.43 (.09) | 1.34 (.08) |  | .89 (.10) | .82 (.09) |  | .68 (.06) | .72 (.06) |
|  |  |  |  |  |  |  |  |  |
| **Controls** |  |  |  |  |  |  |  |  |
| Acquisition (*n* = 42) | 1.79 (.11) | 1.32 (.09) |  | 1.60 (.09) | .93 (.05) |  | 1.54 (.08) | .88 (.08) |
| Extinction (*n* = 41) | 1.57 (.09) | 1.14 (.08) |  | .84 (.07) | .64 (.05) |  | .60 (.06) | .54(.05) |
| Reinstatement (*n* = 40) | 1.36 (.08) | 1.17 (.07) |  | .72 (.07) | .65 (.06) |  | .61 (.06) | .59 (.06) |
|  |  |  |  |  |  |  |  |  |
| **Simulated  Aphantasia** |  |  |  |  |  |  |  |  |
| Acquisition (n = 31) | 1.53 (.08) | 1.22 (.06) |  | 1.36 (.08) | .75 (.06) |  | 1.30 (.09) | .72 (.05) |
| Extinction (n = 26) | 1.40 (.09) | .88 (.07) |  | .83 (.06) | .74 (.07) |  | .79 (.10) | .79 (.08) |
| Reinstatement (n = 25) | 1.39 (.09) | 1.31 (.10) |  | .92 (.11) | .71 (.08) |  | .67 (.06) | .69 (.08) |

*Note.* Standard errors of the mean are displayed in parenthesis. **^1^** Trials within each experimental procedure were partitioned into three bins (start, mid, end; Acquisition & Extinction: trials 1–3, 4–7, 8–10; Reinstatement: trials 1-3, 4-5, 6-8. Skin conductance responses were root-transformed and mean range corrected over all experimental procedures.

## Additional figures

**Figure S1.**

Scatterplots for the correlations between VVIQ score, task specific vividness and BRT priming score in the total sample.


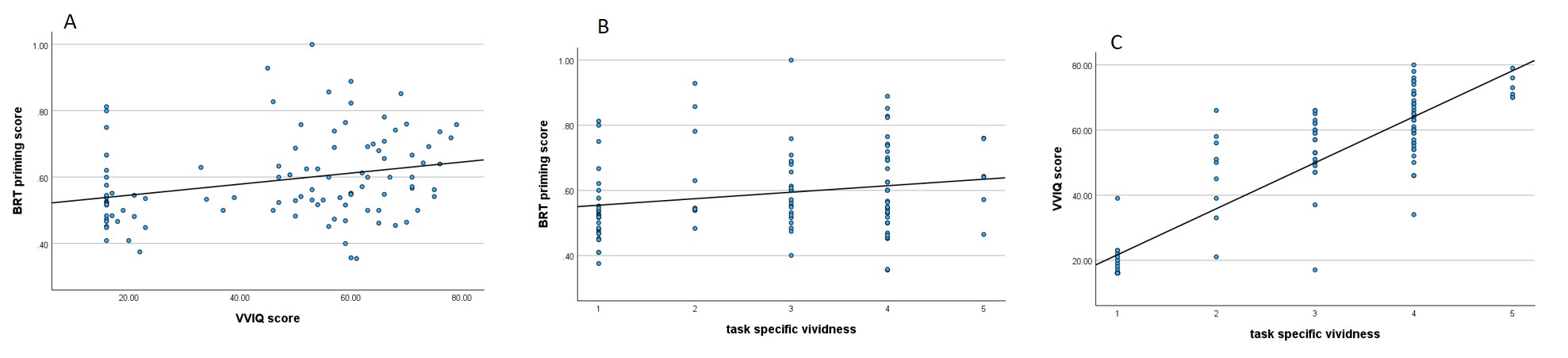


## Reanalyses within the subsample meeting the criteria of compliance and fear acquisition

*Table S2.* Descriptive statistics and tests for differences between groups in sociodemographic variables in the subsample meeting the compliance and fear acquisition criteria.

|  | Aphantasia  (*N* = 16) | Controls  (*N* = 27) | Simulated aphantasia  (*N* = 15) | Test statistic | *p* |
| --- | --- | --- | --- | --- | --- |
| **Age** |  |  |  |  |  |
| *M* | 32.81 | 26.93 | 26.13 |  |  |
| *SD* | 11.27 | 8.24 | 9.08 | 2.55^a^ | .087 |
| **Gender** |  |  |  |  |  |
| Male (%) | 37.5 | 18.5 | 20.0 |  |  |
| Female (%) | 62.5 | 81.5 | 80.0 |  |  |
| Neither/Both (%) | 0.0 | 0.0 | 0.0 | 2.17^b^ | .339 |
| **Handedness** |  |  |  |  |  |
| Left (%) | 28.6 | 0.0 | 13.3 | 3.74^b^ | .154 |
| Right (%) | 71.4 | 100.0 | 86.7 |  |  |
| Both (%) | 0.0 | 0.0 | 0.0 |  |  |

*Note.* ^a^*F*-Test, ^b^ χ^2^-Test.

### Manipulation check: group assignment

Groups differed significantly in the VVIQ sum scores, *F*(2, 55) = 96.89, *p* < .001, η_p_^2^ = .78, *BF_10_* = 3.59×10^15^, the task specific vividness, *F*(2, 54) = 52.45, *p* < .001, η_p_^2^ = .66, *BF_10_* = 2.42×10^10^, as well as the priming score of the binocular rivalry task, *F*(2, 54) = 3.72, *p* = .031, η_p_^2^ = .12, *BF_10_* = 1.94. Post-hoc t-tests revealed that these effects were based on the differences between participants with aphantasia and the other two groups (see Figure S2). Correlational analyses revealed significant associations between the priming score in the binocular rivalry task and the VVIQ, *r*(55) = .35, *p* = .007, *BF_10_* = 5.78, the priming score in the binocular rivalry task and task specific vividness, *r*(54) = .32, *p* = .018, *BF_10_* = 2.75, as well as between the task specific vividness and the VVIQ, *r*(55) = .88, *p* < .001, *BF_10_* = 4.42×10^16^ (see Figure S3).

**Figure S2.**

Differences in visual imagery ability between the groups measured by (a) the vividness of visual imagery questionnaire, (b) task specific vividness and (c) the priming score in the binocular rivalry task. *** p < .001, * p < .05.


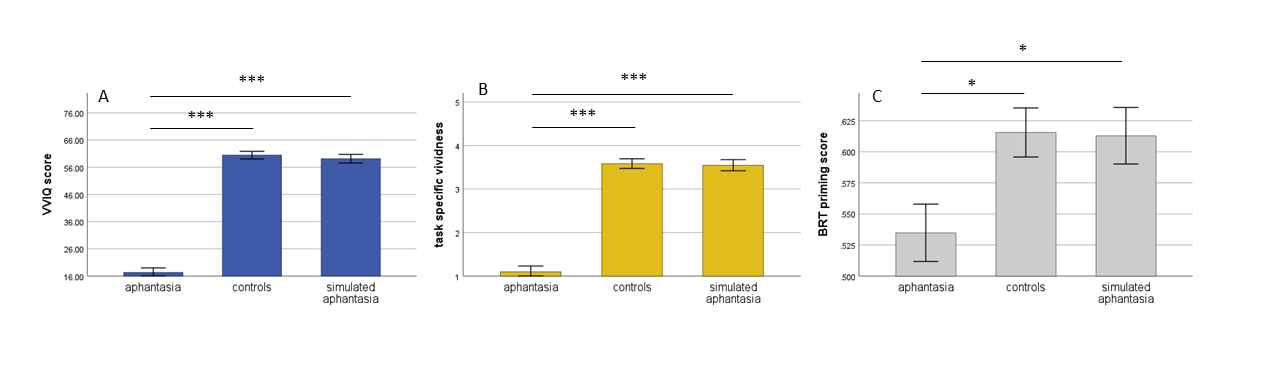


**Figure S3.**

Scatterplots for the correlations between VVIQ score, task specific vividness and BRT priming score.

**
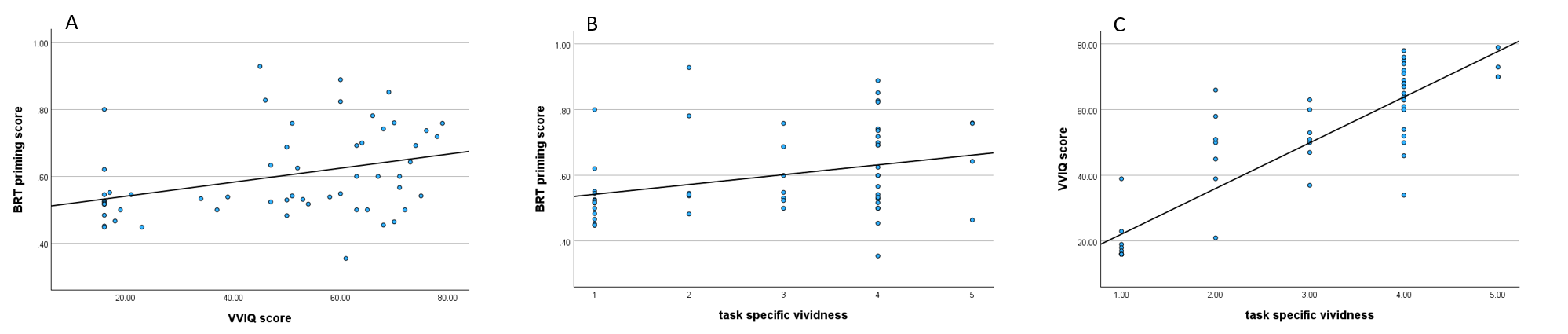
**

### Main analyses: fear extinction

Results showed a significant stimulus x trial interaction effect, *F*(1.78, 85.54) = 23.04, *p* < .001, η_p_^2^ = .32, *BF_10_* = 1.33×10^7^, indicating an overall successful fear extinction procedure. T-tests confirmed that participants differed in response between CS+ and CS- in the beginning, *t*(50) = 6.93, *p* < .001, *d* = 0.97, *BF_10_* = 1.58×10^6^, and middle, *t*(50) = 3.05, *p* = .004, *d* = 0.43, *BF_10_* = 9.04, but not at the end, *t*(50) = 0.52, *p* = .605, *BF_01_* = 5.77. Crucially, there was neither a main effect of group, *F*(2, 48) = 0.30, *p* = .740, η_p_^2^ = .01, *BF_01_* = 7.43, nor a stimulus x group, *F*(2, 48) = 0.62, *p* = .542, η_p_^2^ = .03, *BF_01_* = 7.02, or group x stimulus x trial interaction effect, *F*(3.56, 85.54) = 1.15, *p* = .338, *BF_01_* = 5.04, indicating no differential fear extinction between the groups (see Figure S4). However, there was a trial x group, *F*(2.76, 66.35) = 2.94, *p* = .043, η_p_^2^ = .11, *BF_10_* = 2.68, indicating that the groups differed in general habituation, irrespective of learning. In addition, a significant main effect of stimulus, *F*(1, 48) = 33.75, *p* < .001, η_p_^2^ = .41, *BF_10_* = 14380.66, and a significant main effect of trial were found, *F*(1.38, 66.35) = 49.63, *p* < .001, η_p_^2^ = .51, *BF_10_* = 1.69×10^15^, indicating that the fear response was smaller for the CS- and declined for both the CS+ and CS-. The descriptive statistics for all experimental phases can be found in the Supplemental Material (see Table S3).

**Figure S4.**

Root transformed, mean range corrected SCR for (a) participants with aphantasia, (b) controls and (c) participants in the simulated aphantasia condition across the fear extinction phase (± 1 SEM).


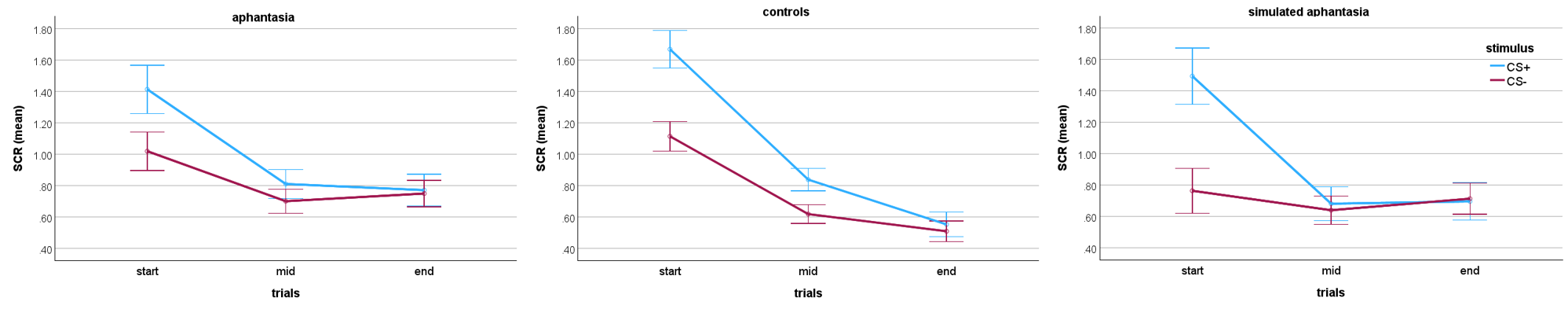


*Table S3.* SCR to the CS+ and CS- across all experimental procedures (subsample).

|  | Trial bin^1^ | | | | | | | |
| --- | --- | --- | --- | --- | --- | --- | --- | --- |
|  | Start | |  | Mid | |  | End | |
|  | CS+ | CS- |  | CS+ | CS- |  | CS+ | CS- |
| **Total** |  |  |  |  |  |  |  |  |
| Acquisition (*n* = 58) | 1.79 (.08) | 1.28 (.07) |  | 1.61(.07) | .83 (.04) |  | 1.57 (.07) | .74 (.03) |
| Extinction (*n* = 51) | 1.56 (.08) | 1.01 (.07) |  | .80 (.05) | .65 (.04) |  | .65 (.06) | .62 (.05) |
| Reinstatement (*n* = 51) | 1.38 (.07) | 1.24 (.07) |  | .80 (.08) | .64 (.05) |  | .63 (.05) | .60 (.05) |
|  |  |  |  |  |  |  |  |  |
| **Aphantasia** |  |  |  |  |  |  |  |  |
| Acquisition (*n* = 16) | 1.67 (.14) | 1.29 (.12) |  | 1.46 (.10) | .80 (.06) |  | 1.44 (.10) | .74 (.06) |
| Extinction (*n* = 15) | 1.41 (.15) | 1.02 (.12) |  | .81 (.09) | .70 (.08) |  | .77 (.07) | .75 (.09) |
| Reinstatement (*n* = 14) | 1.46 (.11) | 1.31 (.12) |  | .86 (.15) | .68 (.13) |  | .66 (.10) | .76 (.10) |
|  |  |  |  |  |  |  |  |  |
| **Controls** |  |  |  |  |  |  |  |  |
| Acquisition (*n* = 27) | 1.96 (.13) | 1.32 (.11) |  | 1.76 (.11) | .87 (.06) |  | 1.70 (.11) | .79 (.05) |
| Extinction (*n* = 25) | 1.65 (.13) | 1.12 (.10) |  | .82 (.08) | .60 (.06) |  | .55 (.08) | .49 (.06) |
| Reinstatement (*n* = 25) | 1.35 (.11) | 1.19 (.11) |  | .67 (.10) | .60 (.07) |  | .57 (.08) | .49 (.07) |
|  |  |  |  |  |  |  |  |  |
| **Simulated  Aphantasia** |  |  |  |  |  |  |  |  |
| Acquisition (n = 15) | 1.61 (.15) | 1.21 (.10) |  | 1.52 (.13) | .78 (.09) |  | 1.47 (.16) | .66 (.07) |
| Extinction (n = 11) | 1.49 (.13) | .76 (.09) |  | .68 (.08) | .64 (.09) |  | .70 (.15) | .71 (.10) |
| Reinstatement (n = 12) | 1.36 (.13) | 1.26 (.16) |  | 1.02 (.19) | .65 (.11) |  | .72 (.10) | .64 (.09) |

*Note.* Standard errors of the mean are displayed in parenthesis. **^1^** Trials within each experimental procedure were partitioned into three bins (start, mid, end; Acquisition & Extinction: trials 1–3, 4–7, 8–10; Reinstatement: trials 1-3, 4-5, 6-8. Skin conductance responses were root-transformed and mean range corrected over all experimental procedures.

### Main analyses: reinstatement

Results showed no stimulus x trial interaction effect, *F*(2, 96) = 1.64, *p* = .200, *BF_01_* = 3.39. However, additional t-tests showed that participants differed in response between CS+ and CS- in the beginning, *t*(50) = 2.01, *p* < .05, *d* = 0.28, *BF_10_* = 0.98, and in the middle, *t*(50) = 2.09, *p* = .042, *d* = 0.29, *BF_10_* = 1.13, but not at the end of the reinstatement, *t*(50) = 0.76, *p* = .452, *BF_01_* = 5.00. Thus, a return of fear effect was noted at the start of the reinstatement, which later faded. There was neither a significant main effect of group, *F*(2, 48) = 1.18, *p* = .316, *BF_01_* = 2.71, nor a trial x group, *F*(3.20, 76.69) = 0.44, *p* = .736, *BF_01_* = 15.73, stimulus x group, *F*(2, 48) = 0.53, *p* = .593, *BF_01_* = 8.15, or group x stimulus x trial interaction effect, *F*(4, 96) = 0.86, *p* = .492, *BF_01_* = 5.88, indicating no differential reinstatement between the groups (see Figure S5). In addition, a significant main effect of stimulus, *F*(1, 48) = 9.78, *p* = .003, η_p_^2^ = .17, *BF_10_* = 3.01, and a significant main effect of trial were found, *F*(1.60, 76.69) = 86.27, *p* < .001, η_p_^2^ = .64, *BF_10_* = 3.41×10^21^, indicating a return of fear effect and that responses for both stimuli declined during the reinstatement procedure.

**Figure S5.**

Root transformed, mean range corrected SCR for (a) participants with aphantasia, (b) controls and (c) participants in the simulated aphantasia condition across the reinstatement phase (± 1 SEM).


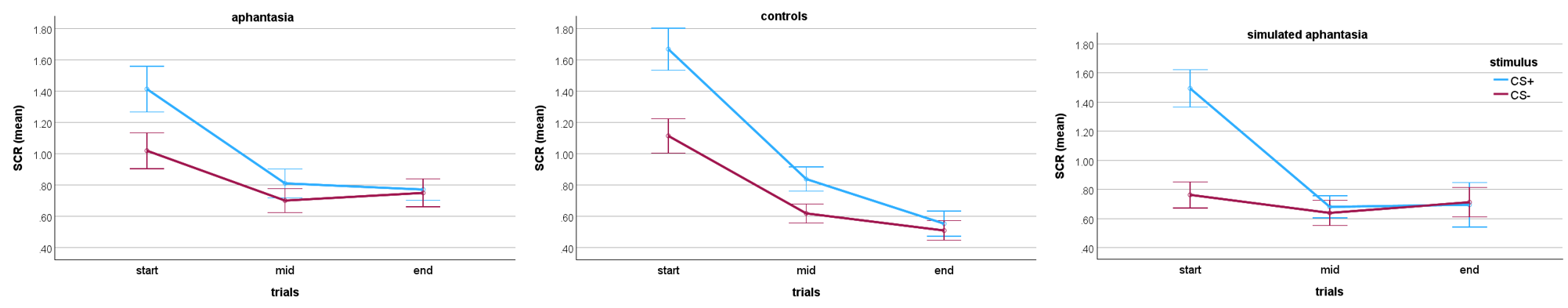


### Trait anxiety, situational fear and shock expectancy

Participants with aphantasia (*M* = 2.06, *SD* = 0.42), controls (*M* = 2.27, *SD* = 0.40) and participants in the simulated aphantasia condition (*M* = 1.98, *SD* = 0.33) did not differ significantly in trait anxiety, *F*(2, 55) = 3.12, *p* = .052, *BF_01_* = 0.70. Regarding the situational fear during the experimental procedure, there was a significant main effect of group, *F*(2, 54) = 4.82, *p* = .012, η_p_^2^ = .15, *BF_10_* = 4.83, indicating that participants with aphantasia (*M* = 10.99, *SD* = 16.97) experienced less fear than controls (*M* = 29.46, *SD* = 19.92), *t*(41) = 3.07, *p_holm_* = .010, *d* = 0.88, *BF_10_* = 2550.21. However, there was no significant difference between participants with aphantasia and participants in the simulated aphantasia condition (*M* = 24.67, *SD* = 16.42), *t*(30) = 2.08, *p_holm_* = .086, *d* = 0.67, *BF_01_* = 0.01, and between controls and participants in the simulated aphantasia condition, *t*(40) = 0.71, *p_holm_* = .483, *d* = 0.21, *BF_01_* = 3.02 (see Figure S6a).^[[1]](#footnote-1)^ Moreover, there was neither a main effect of experimental phase, *F*(2, 108) = 2.12, *p* = .125, *BF_01_* = 2.14, nor an interaction effect between group and experimental phase, *F*(4, 108) = 0.39, *p* = .813, *BF_01_* = 14.01.

An additional repeated-measures ANOVA revealed a significant main effect of experimental phase for shock expectancy, *F*(2, 108) = 46.40, *p* < .001, η_p_^2^ = .46, *BF_10_* = 1.53×10^13^, showing a linear trend, *F*(1, 54) = 81.39, *p* < .001, η_p_^2^ = .60, indicating that the shock expectancy declined across the experimental phases (see Figure S6b). Moreover, there was a main effect of group, *F*(2, 54) = 4.51, *p* = .015, η_p_^2^ = .14, *BF_10_* = 2.47, with controls (*M* = 60.60, *SD* = 15.07) showing higher shock expectancy than participants in the simulated aphantasia condition (*M* = 42.47, *SD* = 14.81), *t*(40) = 2.90, *p_holm_* = .016, *d* = 1.19, *BF_10_* = 108.23, probably due to a higher cognitive load in the simulated aphantasia condition that diverted the attention from the possible shocks. However, there was no difference between the aphantasia and the control group, *t*(40) = 1.80, *p_holm_* = .155, *BF_01_* = 0.92, nor between the aphantasia and the simulated aphantasia group, *t*(40) = 1.03, *p_holm_* = .309, *BF_01_* = 2.67. Moreover, there was no interaction effect between experimental phase and group, *F*(4, 108) = 2.00, *p* = .099, *BF_01_* = 1.65, indicating no different shock expectancy in each experimental phase dependent on group.

**Figure S6.**

(a) Subjective fear ratings and (b) shock expectancies across fear acquisition, fear extinction, and reinstatement per group (± 1 SEM).


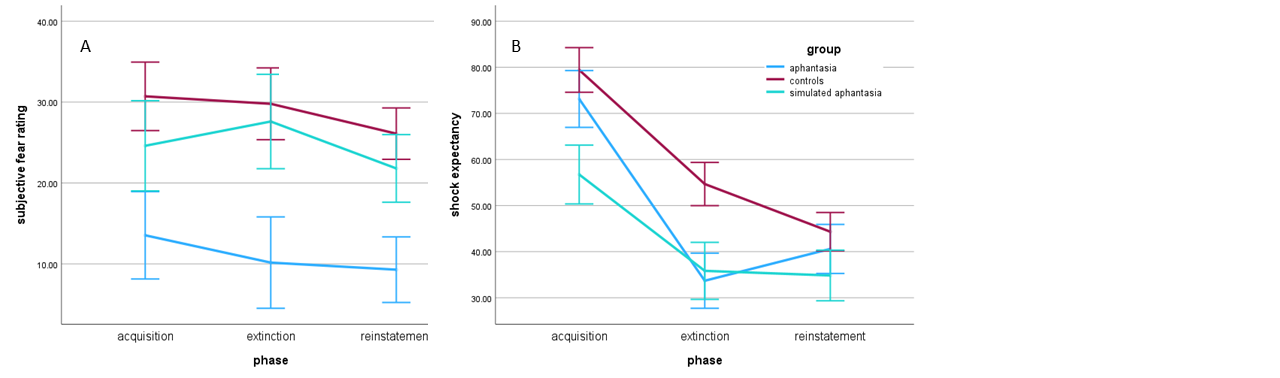


1. The effect remained significant when adding age as a covariate, *F*(2, 53) = 3.63, *p* = .033, η_p_^2^ = .12, *BF_10_* = 2.81. [↑](#footnote-ref-1)
